# Chronic Viral Hepatitis Screening Inequities Across Florida Federally Qualified Health Centers

**DOI:** 10.1007/s40615-025-02363-3

**Published:** 2025-03-06

**Authors:** Hannah M. Cranford, Daniel Parras, Patricia D. Jones, Edelise Endemano, Katherine Chung-Bridges, Paulo S. Pinheiro

**Affiliations:** 1 Division of Epidemiology & Population Health Sciences, Department of Public Health Sciences, University of Miami School of Medicine, Miami, FL USA; 2 Health Choice Network, Miami, FL USA; 3 Division of Digestive Health and Liver Diseases, Department of Medicine, University of Miami School of Medicine, Miami, FL USA; 4 Sylvester Comprehensive Cancer Center, University of Miami Health System, Miami, FL USA

**Keywords:** Screening, Ethnicity, Race, HBV, HCV, Federally qualified health center

## Abstract

**Aim:**

Examine racial and ethnic inequities in hepatitis C and B virus (HCV and HBV) screening across high-risk populations.

**Subject and Methods:**

Chronic HCV and HBV infections can lead to cirrhosis, hepatocellular carcinoma (HCC), and death. Despite universal screening recommendations, < 50% of US adults are tested for these viruses. Populations with lower socio-economic status experience higher rates of viral-related cirrhosis and HCC, which may be reduced through screening and treatment. This study analyzed data from 91,875 patients (2019–2021) at the Health Choice Network, a federally qualified health center in Florida. Logistic regression assessed the determinants of HCV and HBV screening, considering factors such as age, sex, language, and race/ethnicity.

**Results:**

The study population was predominantly female, Hispanic, uninsured, and living below the federal poverty line. Overall, 61.7% had HCV screening, and 43.7% had HBV screening. Haitian Creole-speaking patients (aOR 1.67; 95% CI, 1.50–1.85), Asian (aOR 1.41; 95% CI, 1.26–1.58), Spanish-speaking Hispanic (aOR 1.38; 95% CI, 1.32–1.44), and English-speaking Hispanic patients (aOR 1.12; 95% CI, 1.07–1.18) had higher odds of HCV screening, compared to NH-Whites. Similarly, Haitian Creole-speaking (aOR 1.91; 95% CI, 1.73–2.12), Asian (aOR 1.50; 95% CI, 1.33–1.68), and Spanish-speaking Hispanic patients (aOR 1.23; 95% CI, 1.17–1.29) had higher odds of HBV screening.

**Conclusion:**

For an underserved population context, screening rates were above average, with higher prevalence among historically disadvantaged populations. However, rates remained suboptimal, particularly among NH-Whites, who account for the largest number of HCV-related liver cancers, often linked to injection drug use. Increasing screening, especially among NH-Whites and English-speaking Hispanics for HCV, is crucial for early diagnosis, treatment, and reducing severe liver disease risk, including cirrhosis and HCC.

**Supplementary Information:**

The online version contains supplementary material available at 10.1007/s40615-025-02363-3.

## Introduction

Chronic viral hepatitis infection is a considerable public health concern in the United States (US) with chronic hepatitis C virus (HCV) [1] and chronic hepatitis B virus (HBV) [2] each affecting an estimated 2.4 million people. Untreated, chronic hepatitis infection can lead to serious complications such as cirrhosis, hepatic failure, hepatocellular carcinoma (HCC), and even death [3, 4]. Such infections represent a pervasive and often silent threat to communities, as many infections are asymptomatic [3, 5] and inadvertent transmission can occur from a chronic viral carrier to others.

Screening for HCV and HBV serves as the frontline defense against hepatitis-related liver diseases, offering an opportunity for early detection, intervention, and improved patient outcomes. Recommendations from the Centers for Disease Control and Prevention (CDC) from 2020 and 2023 respectively advise for HCV and HBV screening to be performed in all adults ≥ 18 years old at least once per lifetime [6, 7], equating to a universal screening strategy. Prior to 2020 and 2023, risk-based strategies guided HCV and HBV screening implementation. For HCV, recommendations during 2012–2019 required HCV screening for all people born during 1945–1965 [8], in addition to 1998–2019 recommendations requiring HCV screening for people who have used injection drugs, people with selected medical conditions, and prior recipients of transfusion or organ transplants [9]. For HBV, recommendations during 2008–2022 required HBV screening for people born in countries with an HBsAg prevalence of ≥ 2% (e.g., Haiti, countries in Asia and Africa), close contacts of people infected with HBV, pregnant people, men who have sex with men, and people who use injection drugs [10]. Currently, HCV screening is performed with anti-HCV antibody testing followed by confirmatory polymerase chain reaction testing for HCV RNA [11], while HBV screening is performed using a triple panel test including hepatitis B surface antigen, antibody to hepatitis B surface antigen, and total antibody to hepatitis B core antigen [7]. As chronic hepatitis infection poses a substantial threat to individuals’ well-being, effective and comprehensive HCV and HBV screening practices are important for public health prevention and treatment. Early detection of chronic hepatitis allows for timely treatment initiation—direct-acting antiviral agents (DAAs) for HCV and antiviral agents for HBV. The introduction of DAAs for chronic HCV is of particular importance as treatment allows the achievement of an undetectable sustained virologic response, corresponding to a cure [12].

Despite the availability of reliable screening tools and clinical guidance for screening, there exist concerning inequities in the accessibility, recommendation, and uptake of hepatitis screening across diverse demographic and socio-economic groups. Fewer than half of US adults with HCV or HBV have been screened [13], a large proportion of which consist of people born outside of the US [14–16]. Lower US screening rates for hepatitis have been associated with low socioeconomic status (SES), lack of health insurance, limited provider knowledge, and competing priorities in the clinical setting [17–21]. Nevertheless, preventive screening uptake is strongly correlated with provider recommendation [22, 23]. Previous population-based studies have shown lower HCV screening for women and for non-Hispanic (NH) Black, Asian, and Hispanic populations, compared to NH-White people [20, 24]; in cohort studies, specifically of veterans and patients of community health centers, HCV screening was lower for NH-White people, compared to people of other racial-ethnic groups [25–27]. For HBV, lower screening has also been shown for NH-Black, Asian, and Hispanic populations, compared to NH-White people [17, 20, 28]; studies involving solely minority populations demonstrate higher HBV screening among Asian people, with the lowest among Hispanic people [29, 30].

Goals from the 2023 US Viral Hepatitis National Strategic Plan include screening and diagnosing ≥ 90% of people with HBV and achieving viral clearance in ≥ 80% of people with HCV [31]. However, in Florida, these goals may be stymied by the continued use of risk-factor-based viral hepatitis screening techniques contrary to new universal screening recommendations [32]. Universal HCV and HBV screening and linkage to treatment will help to reduce viral-related liver cancer cases. In Florida, HCV-related HCC cases are higher among Puerto Rican, African American, and US-born Mexican populations and HBV-related HCC cases are higher among Asian and Haitian populations [33]. Yet, the characterization of Florida HCV and HBV screening and potential differences by demographic and socio-economic factors are unclear.

The objective of this analysis is to understand the intricacies of HCV and HBV screening inequities in Florida, particularly among groups that have been historically marginalized in healthcare quality, to help inform the development of effective public health interventions and ultimately, to improve clinical outcomes. This project brought together a team of researchers from Health Choice Network (HCN), a local federally qualified health center (FQHC) cooperative consisting of 25 health centers and 325 primary care delivery sites across the US, University of Miami Miller School of Medicine, and Sylvester Comprehensive Cancer Center. HCN is a vital public healthcare network providing comprehensive primary and preventive care services to diverse medically underserved populations. This study focused on HCN’s Florida health centers.

## Methods

This project utilized individual-level electronic health record (EHR) data on current HCN patients with at least one healthcare visit during 2019–2021. Inclusion criteria for those able to be screened for chronic viral hepatitis were all new and established adult (≥ 18 years of age) HCN patients in Florida. Exclusion criteria include those previously diagnosed with HCV or HBV (ICD-10-CM B18.0, B18.1, B18.2) and those with liver cirrhosis (ICD-10-CM K70.3 and K74.X). Primary outcomes of interest were past receipt of HCV and HBV screening (all years), identified by appropriate laboratory testing (component name) as defined by screening recommendations at the time of the data [9, 10]: for HCV, history of HCV antibody (HCV Ab) test and for HBV, history of HBV surface antigen (HBsAg) test.

Sociodemographic and clinical characteristics of interest included sex, age, primary language and race-ethnicity, health insurance type, employment status, health center county, percentage of federal poverty guideline (FPG), and new versus established patient status. Age in years was categorized into six groups (18–29, 30–39, 40–49, 50–59, 60–69, 70 +) for comparison to previous literature which historically utilizes birth cohort due to previous risk-based screening recommendations for HCV. A combined variable employing primary language, ethnicity, and race was established to help characterize Florida’s large, diverse population (Supplementary Table 1). The primary language was categorized as Haitian Creole, English, Spanish, or other and was used to identify and characterize select Florida populations including patients emanating from Haiti, regardless of race-ethnicity, as nativity is not captured in HCN EHR data. Other groups categorized based on language include Hispanic patients, separated by primary language (Spanish, English, or other language). Therefore, eight mutually exclusive primary language, ethnicity, and race groups were identified in sequence: patients who speak Haitian Creole as a primary language, English-speaking Hispanic patients, Spanish-speaking Hispanic patients, Hispanic patients with another primary language, NH-White patients, NH-Black patients (who do not speak Haitian Creole), Asian patients, and Others. Insurance type was identified by the default insurance available for the patient (Private, Medicaid, Medicare, Uninsured/None). Health center county is the county in which the health center is located and encompasses Miami-Dade, Broward, Orange, Palm Beach, Pasco, and Pinellas. The percentage of FPG was calculated based on US Department of Health and Human Services poverty guidelines [34]. For each patient, the calculated FPG percentage for the most recent year was utilized with patients experiencing the highest poverty meeting 100% and below the FPG and patients experiencing the lowest poverty meeting over 200% of the FPG. HCN patient status was defined as new, a patient with a first-time physical/annual wellness visit during 2019–2021 and, existing, a patient who has completed 2 + physical/annual wellness visits.

Descriptive statistics were used to characterize HCN patients, and comparisons between those screened and those not screened were performed via chi-square tests of independence or Fisher’s exact tests, separately for HCV and HBV. Univariable and multivariable logistic regression was performed testing associations between odds of receipt of HCV and HBV screening and patient characteristics. Model fit was assessed with the Akaike information criterion, and the inclusion of individual explanatory variables was based on the statistical significance of the likelihood-ratio test. The best-fit model for HCV screening, selected based on the lowest AIC (111,719.60), included all covariates without interaction terms. Similarly, the final model for HBV screening had the lowest AIC (110,840.89) and included all covariates without interaction terms. While sex was not statistically significant in the HBV model, it was retained due to its clinical importance. Multivariable model results include adjusted odds ratios (aOR) with 95% confidence intervals (CI). Statistical tests were two-sided and considered significant at *p* < 0.05. Analyses were performed using R Statistical Software (v4.1.2) [35].

## Results

A total of 91,875 HCN patients were included in this analysis (Tables 1 and 2). Patients were majority female (67.7%; *n* = 62,164), Hispanic (50.4%; *n* = 46,346), uninsured (61.4%; *n* = 56,413), and living at or below the FPG (70.1%; *n* = 64,391). The overall prevalence of HCV screening among this cohort was 61.7% (95% CI, 61.3–62.0%), and of HBV screening, was 43.7% (95% CI, 43.4–44.1%).

**Table 1:** Clinical and sociodemographic characteristics of Health Choice Network patients by HCV screening status. Florida, 2019–2021

Variable	Total (*n* (%^a^))	Screened for HCV (*n* (%^a^; %^b^))	Not screened for HCV (*n* (%^a^; %^b^))	*p*-value^c^
**Total (*n* (%**^**b**^**))**	91,875	56,643 (61.7)	35,232 (38.3)	
**Sex**				< 0.001
Female^d^	62,169 (67.7)	37,880 (66.9; 60.9)	24,289 (68.9; 39.1)	
Male	29,706 (32.3)	18,763 (33.1; 63.2)	10,943 (31.1; 36.8)	
**Age group (years)**				< 0.001
18–29	17,442 (19.0)	9684 (17.1; 55.5)	7758 (22.0; 44.5)	
30–39	18,649 (20.3)	11,463 (20.2; 61.5)	7186 (20.4; 38.5)	
40–49	19,421 (21.1)	12,410 (21.9; 63.9)	7011 (19.9; 36.1)	
50–59	20,158 (21.9)	13,033 (23.0; 64.7)	7125 (20.2; 35.3)	
60–69	12,500 (13.6)	8027 (14.2; 64.2)	4473 (12.7; 35.8)	
70 +	3705 (4.0)	2026 (3.6; 54.7)	1679 (4.8; 45.3)	
**Race, ethnicity, and primary language**				< 0.001
NH-White	14,846 (16.2)	6929 (12.2; 46.7)	7917 (22.5; 53.3)	
Haitian Creole Speakers	2257 (2.5)	1664 (2.9; 73.7)	593 (1.7; 26.3)	
English-speaking Hispanic	15,289 (16.6)	9277 (16.4; 60.7)	6012 (17.1; 39.3)	
Spanish-speaking Hispanic	30,899 (33.6)	21,773 (38.4; 70.5)	9126 (25.9; 29.5)	
Other Language Hispanic	158 (0.2)	99 (0.2; 62.7)	59 (0.2; 37.3)	
NH-Black	22,333 (24.3)	13,671 (24.1; 61.2)	8662 (24.6; 38.8)	
Asian	1615 (1.8)	898 (1.6; 55.6)	717 (2; 44.4)	
Other	4478 (4.9)	2332 (4.1; 52.1)	2,146 (6.1; 47.9)	
**Insurance type**				< 0.001
Private	18,550 (20.2)	12,265 (21.7; 66.1)	6285 (17.8; 33.9)	
Medicaid	14,193 (15.4)	7818 (13.8; 55.1)	6375 (18.1; 44.9)	
Medicare	2719 (3.0)	1485 (2.6; 54.6)	1234 (3.5; 45.4)	
Uninsured/none	56,413 (61.4)	35,075 (61.9; 62.2)	21,338 (60.6; 37.8)	
**Employment status**				< 0.001
Full-time	35,692 (38.8)	24,436 (43.1; 68.5)	11,256 (31.9; 31.5)	
Part-time	58 (0.1)	31 (0.1; 53.4)	27 (0.1; 46.6)	
Unemployed	28,252 (30.8)	17,212 (30.4; 60.9)	11,040 (31.3; 39.1)	
Student	2796 (3.0)	1370 (2.4; 49.0)	1426 (4.0; 51)	
Disabled	2804 (3.1)	1502 (2.7; 53.6)	1302 (3.7; 46.4)	
Retired	2582 (2.8)	1379 (2.4; 53.4)	1203 (3.4; 46.6)	
Unreported	19,691 (21.4)	10,713 (18.9; 54.4)	8978 (25.5; 45.6)	
**Health center county**				< 0.001
Miami-Dade	55,698 (60.6)	39,362 (69.5; 70.7)	16,336 (46.4; 29.3)	
Broward	5798 (6.3)	3150 (5.6; 54.3)	2648 (7.5; 45.7)	
Orange	3393 (3.7)	2312 (4.1; 68.1)	1081 (3.1; 31.9)	
Palm Beach	6985 (7.6)	4826 (8.5; 69.1)	2159 (6.1; 30.9)	
Pasco	4907 (5.3)	1676 (3.0; 34.2)	3231 (9.2; 65.8)	
Pinellas	15,094 (16.4)	5317 (9.4; 35.2)	9777 (27.8; 64.8)	
**Percentage of federal poverty guideline**				< 0.001
> 200%	2324 (2.5)	1514 (2.7; 65.1)	810 (2.3; 34.9)	
151–200%	1932 (2.1)	1138 (2.0; 58.9)	794 (2.3; 41.1)	
101–150%	5125 (5.6)	3004 (5.3; 58.6)	2121 (6.0; 41.4)	
≤ 100%	64,391 (70.1)	42,751 (75.5; 66.4)	21,640 (61.4; 33.6)	
Unknown	18,103 (19.7)	8236 (14.5; 45.5)	9867 (28.0; 54.5)	
**Patient status**				< 0.001
Established	13,808 (15.0)	10,216 (18.0; 74.0)	3592 (10.2; 26.0)	
New	78,067 (85.0)	46,427 (82.0; 59.5)	31,640 (89.8; 40.5)	

*HCV* hepatitis C virus, *N* number, *NH* non-Hispanic

^a^Vertical percentage

^b^Horizontal percentage

^c^*p*-value from the chi-square test of independence or Fisher’s exact test

^d^5 unreported sex assumed female

**Table 2:** Clinical and sociodemographic characteristics of Health Choice Network patients by HBV screening status. Florida, 2019–2021

Variable	Total (*n* (%^a^))	Screened for HBV (*n* (%^a^; %^b^))	Not screened for HBV (*n* (%^a^; %^b^))	*p*-value^c^
**Total (*n* (%**^**b**^**))**	91,875	40,190 (43.7)	51,685 (56.3)	
**Sex**				< 0.001
Female^d^	62,169 (67.7)	27,410 (68.2; 44.1)	34,759 (67.3; 55.9)	
Male	29,706 (32.3)	12,780 (31.8; 43.0)	16,926 (32.7; 57.0)	
**Age group (years)**				< 0.001
18–29	17,442 (19.0)	7564 (18.8; 43.4)	9878 (19.1; 56.6)	
30–39	18,649 (20.3)	8922 (22.2; 47.8)	9727 (18.8; 52.2)	
40–49	19,421 (21.1)	8682 (21.6; 44.7)	10,739 (20.8; 55.3)	
50–59	20,158 (21.9)	8622 (21.5; 42.8)	11,536 (22.3; 57.2)	
60–69	12,500 (13.6)	5214 (13.0; 41.7)	7286 (14.1; 58.3)	
70 +	3705 (4.0)	1186 (3.0; 32.0)	2519 (4.9; 68.0)	
**Race, ethnicity, and primary language**				< 0.001
NH-White	14,846 (16.2)	5013 (12.5; 33.8)	9833 (19.0; 66.2)	
Haitian Creole Speakers	2257 (2.5)	1180 (2.9; 52.3)	1077 (2.1; 47.7)	
English-speaking Hispanic	15,289 (16.6)	5832 (14.5; 38.1)	9457 (18.3; 61.9)	
Spanish-speaking Hispanic	30,899 (33.6)	16,084 (40.0; 52.1)	14,815 (28.7; 47.9)	
Other Language Hispanic	158 (0.2)	81 (0.2; 51.3)	77 (0.1; 48.7)	
NH-Black	22,333 (24.3)	10,011 (24.9; 44.8)	12,322 (23.8; 55.2)	
Asian	1615 (1.8)	676 (1.7; 41.9)	939 (1.8; 58.1)	
Other	4478 (4.9)	1313 (3.3; 29.3)	3165 (6.1; 70.7)	
**Insurance type**				< 0.001
Private	18,550 (20.2)	9111 (22.7; 49.1)	9439 (18.3; 50.9)	
Medicaid	14,193 (15.4)	5790 (14.4; 40.8)	8403 (16.3; 59.2)	
Medicare	2719 (3.0)	729 (1.8; 26.8)	1990 (3.9; 73.2)	
Uninsured/none	56,413 (61.4)	24,560 (61.1; 43.5)	31,853 (61.6; 56.5)	
**Employment status**				< 0.001
Full-time	35,692 (38.8)	20,497 (51.0; 57.4)	15,195 (29.4; 42.6)	
Part-time	58 (0.1)	5 (0.0; 8.6)	53 (0.1; 91.4)	
Unemployed	28,252 (30.8)	12,178 (30.3; 43.1)	16,074 (31.1; 56.9)	
Student	2796 (3.0)	978 (2.4; 35.0)	1818 (3.5; 65.0)	
Disabled	2804 (3.1)	885 (2.2; 31.6)	1919 (3.7; 68.4)	
Retired	2582 (2.8)	918 (2.3; 35.6)	1664 (3.2; 64.4)	
Unreported	19,691 (21.4)	4729 (11.8; 24)	14,962 (28.9; 76.0)	
**Health center county**				< 0.001
Miami-Dade	55,698 (60.6)	29,985 (74.6; 53.8)	25,713 (49.7; 46.2)	
Broward	5798 (6.3)	2805 (7.0; 48.4)	2993 (5.8; 51.6)	
Orange	3393 (3.7)	795 (2.0; 23.4)	2598 (5.0; 76.6)	
Palm Beach	6985 (7.6)	1211 (3.0; 17.3)	5774 (11.2; 82.7)	
Pasco	4907 (5.3)	980 (2.4; 20.0)	3927 (7.6; 80.0)	
Pinellas	15,094 (16.4)	4414 (11.0; 29.2)	10,680 (20.7; 70.8)	
**Percentage of federal poverty guideline**				< 0.001
> 200%	2324 (2.5)	1064 (2.6; 45.8)	1260 (2.4; 54.2)	
151–200%	1932 (2.1)	724 (1.8; 37.5)	1208 (2.3; 62.5)	
101–150%	5125 (5.6)	1935 (4.8; 37.8)	3190 (6.2; 62.2)	
≤ 100%	64,391 (70.1)	31,617 (78.7; 49.1)	32,774 (63.4; 50.9)	
Unknown	18,103 (19.7)	4850 (12.1; 26.8)	13,253 (25.6; 73.2)	
**Patient status**				< 0.001
Established	13,808 (15.0)	7192 (17.9; 52.1)	6616 (12.8; 47.9)	
New	78,067 (85.0)	32,998 (82.1; 42.3)	45,069 (87.2; 57.7)	

*HBV* hepatitis B virus, *N* number, *NH* non-Hispanic

^a^Vertical percentage

^b^Horizontal percentage

^c^*p*-value from the chi-square test of independence or Fisher’s exact test

^d^5 unreported sex assumed female

### HCV Screening

Univariable logistic regression (Table 3) displayed males with 10% higher odds of HCV screening, compared to females. Unemployed individuals had 28% lower odds of HCV screening and students had 56% lower odds, compared to those with full-time employment.

**Table 3:** Determinants of HCV and HBV screening among Health Choice Network patients displayed by odds ratios and 95% confidence intervals. Florida, 2019–2021 (*N* = 91,875)

Variable	HCV screening			HBV screening		
	Univariable OR (95% CI)	Multivariable OR (95% CI)	*p***-**value^a^	Univariable OR (95% CI)	Multivariable OR (95% CI)	*p***-**value^a^
**Sex**						
Female	1.00 (Reference)	1.00 (Reference)	-	1.00 (Reference)	1.00 (Reference)	-
Male	1.10 (1.07, 1.13)	1.08 (1.05, 1.11)	< 0.001	0.96 (0.93,0.98)	0.99 (0.96,1.03)	0.686
**Age group (years)**						
18–29	1.00 (Reference)	1.00 (Reference)	-	1.00 (Reference)	1.00 (Reference)	-
30–39	1.28 (1.23, 1.33)	1.20 (1.14, 1.25)	< 0.001	1.20 (1.15, 1.25)	1.07 (1.02, 1.12)	0.007
40–49	1.42 (1.36, 1.48)	1.23 (1.17, 1.29)	< 0.001	1.06 (1.01, 1.10)	0.87 (0.83, 0.91)	< 0.001
50–59	1.47 (1.41, 1.53)	1.25 (1.19, 1.31)	< 0.001	0.98 (0.94, 1.02)	0.79 (0.76, 0.83)	< 0.001
60–69	1.44 (1.37, 1.51)	1.27 (1.20, 1.34)	< 0.001	0.93 (0.89, 0.98)	0.80 (0.76, 0.85)	< 0.001
70 +	0.97 (0.90, 1.04)	0.84 (0.77, 0.91)	< 0.001	0.61 (0.57, 0.66)	0.55 (0.51, 0.60)	< 0.001
**Race, ethnicity, and primary language**						
NH-White	1.00 (Reference)	1.00 (Reference)	-	1.00 (Reference)	1.00 (Reference)	-
Haitian Creole Speakers	3.21 (2.90, 3.54)	1.67 (1.50, 1.85)	< 0.001	2.15 (1.97, 2.35)	1.91 (1.73, 2.12)	< 0.001
English Speaking Hispanic	1.76 (1.68, 1.85)	1.12 (1.07, 1.18)	< 0.001	1.21 (1.15, 1.27)	0.87 (0.83, 0.92)	< 0.001
Spanish Speaking Hispanic	2.73 (2.62, 2.84)	1.38 (1.32, 1.44)	< 0.001	2.13 (2.04, 2.22)	1.23 (1.17, 1.29)	< 0.001
Other Language Hispanic	1.92 (1.39, 2.65)	1.22 (0.87, 1.72)	0.245	2.06 (1.51, 2.82)	1.36 (0.97, 1.91)	0.079
NH-Black	1.80 (1.73, 1.88)	1.17 (1.12, 1.23)	< 0.001	1.59 (1.53, 1.66)	1.13 (1.07, 1.18)	< 0.001
Asian	1.43 (1.29, 1.59)	1.41 (1.26, 1.58)	< 0.001	1.41 (1.27, 1.57)	1.50 (1.33, 1.68)	< 0.001
Other	1.24 (1.16, 1.33)	1.10 (1.03, 1.18)	0.008	0.81 (0.76,0.88)	1.00 (0.92,1.08)	0.965
**Insurance type**						
Private	1.00 (Reference)	1.00 (Reference)	-	1.00 (Reference)	1.00 (Reference)	-
Medicaid	0.63 (0.60, 0.66)	1.02 (0.97, 1.07)	0.433	0.71 (0.68, 0.75)	1.05 (1.00, 1.10)	0.057
Medicare	0.62 (0.57, 0.67)	0.77 (0.70, 0.84)	< 0.001	0.38 (0.35, 0.42)	0.55 (0.50, 0.61)	< 0.001
Uninsured	0.84 (0.81, 0.87)	0.78 (0.75, 0.81)	< 0.001	0.80 (0.77, 0.83)	0.99 (0.95, 1.03)	0.548
**Employment status**						
Full-time	1.00 (Reference)	1.00 (Reference)	-	1.00 (Reference)	1.00 (Reference)	-
Part-time	0.53 (0.32, 0.89)	0.41 (0.25, 0.70)	< 0.001	0.07 (0.03, 0.17)	0.06 (0.02, 0.15)	< 0.001
Unemployed	0.72 (0.70, 0.74)	0.75 (0.72, 0.78)	< 0.001	0.56 (0.54, 0.58)	0.62 (0.60, 0.64)	< 0.001
Student	0.44 (0.41, 0.48)	0.60 (0.55, 0.66)	< 0.001	0.40 (0.37, 0.43)	0.42 (0.39, 0.46)	< 0.001
Disabled	0.53 (0.49, 0.57)	0.73 (0.67, 0.80)	< 0.001	0.34 (0.31, 0.37)	0.49 (0.45, 0.54)	< 0.001
Retired	0.53 (0.49, 0.57)	0.73 (0.66, 0.80)	< 0.001	0.41 (0.38, 0.44)	0.64 (0.58, 0.71)	< 0.001
Unreported	0.55 (0.53, 0.57)	0.65 (0.62, 0.68)	< 0.001	0.23 (0.23, 0.24)	0.22 (0.21, 0.23)	< 0.001
**Health center county**						
Miami-Dade	1.00 (Reference)	1.00 (Reference)	-	1.00 (Reference)	1.00 (Reference)	-
Broward	0.49 (0.47, 0.52)	0.62 (0.58, 0.66)	< 0.001	0.80 (0.76, 0.85)	2.16 (2.01, 2.31)	< 0.001
Orange	0.89 (0.82, 0.96)	0.96 (0.89, 1.04)	0.353	0.26 (0.24, 0.28)	0.24 (0.22, 0.26)	< 0.001
Palm Beach	0.93 (0.88, 0.98)	1.28 (1.20, 1.36)	< 0.001	0.18 (0.17, 0.19)	0.40 (0.37, 0.43)	< 0.001
Pasco	0.22 (0.20, 0.23)	0.27 (0.25, 0.29)	< 0.001	0.21 (0.20, 0.23)	0.23 (0.21, 0.24)	< 0.001
Pinellas	0.23 (0.22, 0.23)	0.31 (0.30, 0.33)	< 0.001	0.35 (0.34, 0.37)	0.67 (0.64, 0.70)	< 0.001
**Percentage of federal poverty guideline**						
> 200%	1.00 (Reference)	1.00 (Reference)	-	1.00 (Reference)	1.00 (Reference)	-
151–200%	0.77 (0.68, 0.87)	0.93 (0.82, 1.06)	0.292	0.71 (0.63, 0.80)	0.83 (0.73, 0.95)	0.005
101–150%	0.76 (0.68, 0.84)	0.88 (0.79, 0.97)	0.015	0.72 (0.65, 0.79)	0.83 (0.75, 0.92)	< 0.001
≤ 100%	1.06 (0.97, 1.15)	1.12 (1.03, 1.23)	0.013	1.14 (1.05, 1.24)	1.31 (1.20, 1.44)	< 0.001
Unknown	0.45 (0.41, 0.49)	0.76 (0.69, 0.84)	< 0.001	0.43 (0.40, 0.47)	0.60 (0.54, 0.66)	< 0.001
**Patient status**						
Established	1.00 (Reference)	1.00 (Reference)	-	1.00 (Reference)	1.00 (Reference)	-
New	0.52 (0.50, 0.54)	0.59 (0.57, 0.62)	< 0.001	0.67 (0.65, 0.70)	0.71 (0.69, 0.74)	< 0.001

*CI* confidence interval, *HBV* hepatitis B virus, *HCV* hepatitis C virus, *NH* non-Hispanic, *OR* odds ratio

^a^p-value from the Wald test for multivariable

**Fig. 1:**
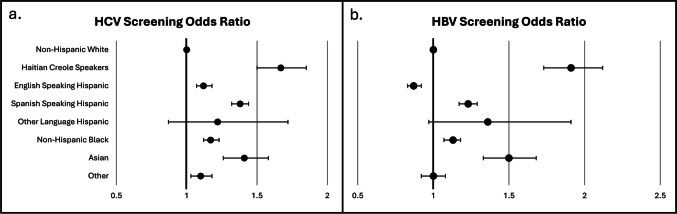
Effect of race, ethnicity, and primary language on HCV (**a**) and HBV (**b**) screening for Health Choice Network patients displayed by odds ratios and 95% confidence intervals, Florida, 2019–2021. Odds ratios are adjusted for clinical and socio-demographic factors and expressed with non-Hispanic White patients as reference

In multivariable logistic regression, controlling for clinical and socio-demographic factors and compared to NH-White patients, the odds of HCV screening were higher for the following groups analyzed: Haitian Creole-speaking patients at 1.67 (95% CI, 1.50–1.85), NH-Asian patients at 1.41 (95% CI, 1.26–1.58), Spanish-speaking Hispanic patients at 1.38 (95% CI, 1.32–1.44), NH-Black patients at 1.17 (95% CI, 1.12–1.23), English-speaking Hispanic patients at 1.12 (95% CI, 1.07–1.18), and Others at 1.10 (95% CI, 1.03–1.18) (Fig. 1). Hispanic patients with another primary language did not have significantly different odds of HCV screening, compared to NH-White patients. Patients with Medicare had 23% lower odds of HCV screening and uninsured patients, 22% lower, compared to patients with private insurance. There was ample variation by county ranging from Palm Beach with 28% higher odds of HCV screening to Pasco with 73% lower odds, compared to Miami-Dade. Older patients (70 +) had significantly lower odds of HCV screening, compared to young patients (18–29). Patients living at or below the FPG had 12% higher odds of HCV screening, compared to patients living over 200% above the FPG. Furthermore, new patients had 41% lower odds of HCV screening, compared to established patients.

### HBV Screening

Univariable modeling showed older patients (70 years and older) with 39% lower odds of HBV screening, compared to young patients (aged 18–29). Differences in patient employment status, compared to full-time patients, ranged from unemployed patients with 44% lower odds of HBV screening to part-time patients with 93% lower odds.

Adjusted odds of HBV screening, compared to NH-White patients, were higher for the following groups analyzed: Haitian Creole-speaking patients at 1.91 (95% CI, 1.73–2.12), Asian patients at 1.50 (95% CI, 1.33–1.68), and Spanish-speaking Hispanic patients at 1.23 (95% CI, 1.17–1.29). English-speaking Hispanic patients had 13% lower odds of HBV screening (aOR 0.87; 95% CI, 0.83–0.92), compared to NH-White patients. Hispanic patients with another primary language and Others did not have significantly different odds of HBV screening, compared to NH-White patients. Patients with Medicare had 45% lower odds of HBV screening, compared to patients with private insurance. Differences by county ranged from Broward with 2.16 times higher odds of HBV screening to Pasco with 77% lower odds, compared to Miami-Dade. Patients living at or below the FPG had 31% higher odds of HBV screening, compared to patients living over 200% above the FPG. Lastly, new patients had 29% lower odds of HBV screening, compared to established patients.

## Discussion

This analysis reveals a notably high prevalence of hepatitis screening among FQHC patients in Florida, with over 60% screened for HCV and nearly 44% for HBV. These proportions far surpass previous US community health center hepatitis screening rates, which were typically at or below 10% [25, 27, 36]. Notably, much of this screening occurred before the CDC's universal screening recommendations for HCV (2020) and HBV (2023), suggesting that Florida FQHCs were already leading the way in implementing comprehensive hepatitis screening practices.

HCV screening rates among Haitian Creole-speaking patients at HCN exceed previous findings showing significantly lower screening rates for US Haitian populations compared to other racial-ethnic groups [27]. Similarly, HBV screening rates for Haitian Creole-speaking patients reflect the effective implementation of the CDC’s 2008–2022 risk-based recommendations, which targeted individuals born in regions with high HBV prevalence [10], such as Haiti [37]. These findings illustrate the positive impact of targeted recommendations on vulnerable populations despite ongoing barriers to care and historical disparities.

Conversely, lower HCV and HBV screening rates among NH-White and English-speaking Hispanic patients are concerning. The NH-White population, particularly those from lower socioeconomic backgrounds, represents the largest absolute number of HCV-related HCC cases [33, 38], largely driven by risk factors like injection drug use [39]. Similarly, increasing HCV screening among English-speaking Hispanic patients is critical, as US-born Hispanics—often English speakers—exhibit the highest age-adjusted incidence rates for HCV-related HCC [33, 40]. Early screening and treatment in these groups are essential to reduce liver disease burden and curb transmission.

Another persistent inequity of interest is insurance status for which differences in hepatitis screening are evident for patients with Medicare. Lower viral hepatitis screening rates for Medicare patients are likely intertwined with Medicare coverage of screening tests. Despite new universal screening recommendations, Medicare only covers HCV screening for people who were born during 1945–1965, have had a blood transfusion before 1992, or are at high risk due to use of illicit injection drugs and only covers HBV screening for people at high risk or who are pregnant [41]. To align with the CDC’s recommendations for universal hepatitis screening and ensure broader access to early detection and treatment, Medicare policy should be updated to include coverage for hepatitis C and B screening for all patients, regardless of risk factors. Considering that over 85% of all Medicare beneficiaries are age 65 or older [42], overlapping largely with HCV screening coverage for people born during 1945–1965, our study shows that the prevalence of HCV screening among HCN patients with Medicare is lower than expected and is an important area of improvement for preventive services.

County-level differences in screening also emerged, with Palm Beach HCN patients showing higher HCV screening rates and Broward County patients having more than double the odds of HBV screening compared to Miami-Dade County. These variations may reflect differences in provider practices, time constraints, and competing demands [19, 21, 43], particularly before the adoption of universal screening. Addressing these disparities will require further research and targeted interventions, including provider education, streamlined screening protocols, and a focus on screening new patient populations.

Overall, this study underscores significant progress in hepatitis screening among historically marginalized populations, particularly Haitian Creole-speaking and Spanish-speaking Hispanic patients, who face barriers to healthcare in general [44, 45]. These higher screening rates possibly reflect improvements in addressing long-standing systemic inequities. This success is likely driven by HCN’s proactive organizational strategies, including provider education through webinars, regular engagement with health centers, and structured feedback mechanisms. Such efforts enable health centers to align with the US Viral Hepatitis National Strategic Plan goals and Health Resources and Services Administration’s Uniform Data System Clinical Quality Measures [46], marking a critical step toward equitable healthcare delivery.

This study is not without limitations, as data on HBV vaccination status, education level, patient residence (urban versus rural), and provider-level factors was unavailable. Some misclassification due to incomplete data in certain covariates, such as employment and income, is possible. However, this does not diminish the study’s importance in uncovering nuanced differences across diverse populations. While the findings are limited to Florida FQHC patients and do not account for potential disruptions from the COVID-19 pandemic, they serve as a valuable foundation for addressing hepatitis screening disparities particularly among groups who are seldom studied (e.g., the US Haitian community, English-speaking Hispanics, etc.). Areas of future research include the assessment of the long-term impact of the CDC universal hepatitis screening guidelines on screening rates and related health outcomes such as liver disease, cirrhosis, and hepatocellular carcinoma, with emphasis on highly impacted populations. To enhance generalizability, similar studies should be performed at a population level.

In summary, this study underscores the essential role of hepatitis screening protocols in Florida FQHCs, particularly for historically marginalized populations such as Haitian Creole-speaking and Spanish-speaking Hispanic patients. While progress toward US Viral Hepatitis National Strategic Plan goals is evident, screening remains uneven. Universal screening implementation is vital for populations with large numbers of HCV-related liver disease including NH-White patients and English-speaking Hispanics experiencing higher poverty. Understanding and addressing the factors driving these disparities will enable more equitable screening practices, ensuring timely diagnosis and treatment to mitigate severe liver disease.

## Supplementary Information

Below is the link to the electronic supplementary material.

Supplementary file1 (DOCX 17 KB)

## Data Availability

The data analyzed during the current study are not publicly available due to privacy, but are available from Health Choice Network on reasonable request.

## References

[1] Hofmeister MG, Rosenthal EM, Barker LK, Rosenberg ES, Barranco MA, Hall EW, Edlin BR, Mermin J, Ward JW, Ryerson AB. Estimating prevalence of hepatitis C virus infection in the United States, 2013–2016. Hepatology. 2019;69(3):1020–31. 10.1002/hep.30297. 30398671 10.1002/hep.30297 PMC6719781

[2] Wong RJ, Brosgart CL, Welch S, Block T, Chen M, Cohen C, Kim WR, Kowdley KV, Lok AS, Tsai N, et al. An updated assessment of chronic hepatitis B prevalence among foreign-born persons living in the United States. Hepatology. 2021;74(2):607–26. 10.1002/hep.31782. 33655536 10.1002/hep.31782 PMC8453838

[3] Fattovich G, Bortolotti F, Donato F. Natural history of chronic hepatitis B: special emphasis on disease progression and prognostic factors. J Hepatol. 2008;48(2):335–52. 10.1016/j.jhep.2007.11.011. 18096267 10.1016/j.jhep.2007.11.011

[4] Liang TJ, Rehermann B, Seeff LB, Hoofnagle JH. Pathogenesis, natural history, treatment, and prevention of hepatitis C. Ann Intern Med. 2000;132(4):296–305. 10.7326/0003-4819-132-4-200002150-00008. 10681285 10.7326/0003-4819-132-4-200002150-00008

[5] Odenwald MA, Paul S. Viral hepatitis: past, present, and future. World J Gastroenterol. 2022;28(14):1405–29. 10.3748/wjg.v28.i14.1405. 35582678 10.3748/wjg.v28.i14.1405 PMC9048475

[6] Schillie S, Wester C, Osborne M, Wesolowski L, Ryerson AB. CDC recommendations for hepatitis C screening among adults - United States, 2020. MMWR Recomm Rep. 2020;69(2):1–17. 10.15585/mmwr.rr6902a1. 32271723 10.15585/mmwr.rr6902a1 PMC7147910

[7] Conners EE, Panagiotakopoulos L, Hofmeister MG, Spradling PR, Hagan LM, Harris AM, Rogers-Brown JS, Wester C. Nelson NP 2023 Screening and testing for hepatitis B virus infection: CDC recommendations - United States. MMWR Recomm Rep. 2023;72(1):1–25. 10.15585/mmwr.rr7201a1. 36893044 10.15585/mmwr.rr7201a1 PMC9997714

[8] Smith BD, Morgan RL, Beckett GA, Falck-Ytter Y, Holtzman D, Teo CG, Jewett A, Baack B, Rein DB, Patel N, et al. Recommendations for the identification of chronic hepatitis C virus infection among persons born during 1945–1965. MMWR Recomm Rep. 2012;61:1–32. 22895429

[9] Centers for Disease Control and Prevention. Recommendations for prevention and control of hepatitis C virus (HCV) infection and HCV-related chronic disease. MMWR Recomm Rep. 1998;1998(47):1–39. 9790221

[10] Weinbaum CM, Williams I, Mast EE, Wang SA, Finelli L, Wasley A, Neitzel SM, Ward JW. Recommendations for identification and public health management of persons with chronic hepatitis B virus infection. MMWR Recomm Rep. 2008;57:1–20. 18802412

[11] Cartwright EJ, Patel P, Kamili S, Wester C. Updated operational guidance for implementing CDC’s recommendations on testing for hepatitis C virus infection. MMWR Morb Mortal Wkly Rep. 2023;72(28):766–8. 10.15585/mmwr.mm7228a2. 37440452 10.15585/mmwr.mm7228a2 PMC10360608

[12] Ioannou GN, Green PK, Berry K. HCV eradication induced by direct-acting antiviral agents reduces the risk of hepatocellular carcinoma. J Hepatol. 2017;68:25. 10.1016/j.jhep.2017.08.030. 10.1016/j.jhep.2017.08.030 PMC5837901 28887168

[13] Zhou K, Terrault NA. Gaps in viral hepatitis awareness in the United States in a population-based study. Clin Gastroenterol Hepatol. 2020;18(1):188-195.e184. 10.1016/j.cgh.2019.05.047. 31173892 10.1016/j.cgh.2019.05.047 PMC8028744

[14] Roberts H, Ly KN, Yin S, Hughes E, Teshale E, Jiles R. Prevalence of HBV infection, vaccine-induced immunity, and susceptibility among at-risk populations: US households, 2013–2018. Hepatology. 2021;74(5):2353–65. 10.1002/hep.31991. 34097776 10.1002/hep.31991

[15] Razavi-Shearer D, Gamkrelidze I, Pan CQ, Razavi-Shearer K, Blach S, Estes C, Mooneyhan E, Razavi H. The impact of immigration on hepatitis B burden in the United States: a modelling study. Lancet Reg Health Am. 2023;22:100516. 10.1016/j.lana.2023.100516. 37274551 10.1016/j.lana.2023.100516 PMC10239007

[16] Gnanapandithan K, Ghali MP. Self-awareness of hepatitis C infection in the United States: a cross-sectional study based on the National Health Nutrition and Examination Survey. PLoS ONE. 2023;18(10):e0293315. 10.1371/journal.pone.0293315. 37874815 10.1371/journal.pone.0293315 PMC10597475

[17] Chu JN, Nguyen TT, Rivadeneira NA, Hiatt RA, Sarkar U. Exploring factors associated with hepatitis B screening in a multilingual and diverse population. BMC Health Serv Res. 2022;22(1):479. 10.1186/s12913-022-07813-w. 35410249 10.1186/s12913-022-07813-w PMC8996655

[18] Chak EW, Sarkar S, Bowlus C. Improving Healthcare systems to reduce healthcare disparities in viral hepatitis. Dig Dis Sci. 2016;61(10):2776–83. 10.1007/s10620-016-4205-3. 27234269 10.1007/s10620-016-4205-3

[19] Kasting ML, Rathwell J, Gabhart KM, Garcia J, Roetzheim RG, Carrasquillo O, Giuliano AR, Vadaparampil ST. There’s just not enough time: a mixed methods pilot study of hepatitis C virus screening among baby boomers in primary care. BMC Fam Pract. 2020;21(1):248. 10.1186/s12875-020-01327-2. 33267799 10.1186/s12875-020-01327-2 PMC7713319

[20] Kim HS, Yang JD, El-Serag HB, Kanwal F. Awareness of chronic viral hepatitis in the United States: an update from the National Health and Nutrition Examination Survey. J Viral Hepat. 2019;26(5):596–602. 10.1111/jvh.13060. 30629790 10.1111/jvh.13060

[21] Mahfouz M, Nguyen H, Tu J, Diaz CR, Anjan S, Brown S, Bosire K, Carrasquillo O, Martin P, Jones PD. Knowledge and perceptions of hepatitis B and hepatocellular carcinoma screening guidelines among trainees: a tale of three centers. Dig Dis Sci. 2020;65(9):2551–61. 10.1007/s10620-019-05980-1. 31813133 10.1007/s10620-019-05980-1

[22] Finney Rutten LJ, Nelson DE, Meissner HI. Examination of population-wide trends in barriers to cancer screening from a diffusion of innovation perspective (1987–2000). Prev Med. 2004;38(3):258–68. 10.1016/j.ypmed.2003.10.011. 14766107 10.1016/j.ypmed.2003.10.011

[23] Peterson EB, Ostroff JS, DuHamel KN, D’Agostino TA, Hernandez M, Canzona MR, Bylund CL. Impact of provider-patient communication on cancer screening adherence: a systematic review. Prev Med. 2016;93:96–105. 10.1016/j.ypmed.2016.09.034. 27687535 10.1016/j.ypmed.2016.09.034 PMC5518612

[24] Kasting ML, Giuliano AR, Reich RR, Roetzheim RG, Nelson DR, Shenkman E, Vadaparampil ST. Hepatitis C virus screening trends: serial cross-sectional analysis of the National Health Interview Survey population, 2013–2015. Cancer Epidemiol Biomarkers Prev. 2018;27(4):503–13. 10.1158/1055-9965.Epi-17-0855. 29588306 10.1158/1055-9965.EPI-17-0855 PMC5884715

[25] Assoumou SA, Wang J, Nolen S, Eftekhari Yazdi G, Mayer KH, Puro J, Salomon JA, Linas BP. HCV testing and treatment in a national sample of US federally qualified health centers during the opioid epidemic. J Gen Intern Med. 2020;35(5):1477–83. 10.1007/s11606-020-05701-9. 32133577 10.1007/s11606-020-05701-9 PMC7210368

[26] Backus LI, Belperio PS, Loomis TP, Mole LA. Impact of race/ethnicity and gender on HCV screening and prevalence among US veterans in Department of Veterans Affairs Care. Am J Public Health. 2014;104(Suppl 4):S555-561. 10.2105/ajph.2014.302090. 25100421 10.2105/AJPH.2014.302090 PMC4151890

[27] Cook N, Turse EP, Garcia AS, Hardigan P, Amofah SA. Hepatitis C virus infection screening within community health centers. J Am Osteopath Assoc. 2016;116(1):6–11. 10.7556/jaoa.2016.001. 26745559 10.7556/jaoa.2016.001

[28] Wong RJ, Campbell B, Liu B, Baden R, Bhuket T. Sub-optimal testing and awareness of HCV and HBV among high risk individuals at an underserved safety-net hospital. J Community Health. 2018;43(1):65–9. 10.1007/s10900-017-0388-6. 28647860 10.1007/s10900-017-0388-6

[29] Begum TF, Patil VS, Zhu L, Yeh MC, González E, Fraser MA, Lu W, Zhu S, Rubio-Torio N, Ma GX, et al. Assessing physicians’ recommendations for hepatitis B virus (HBV) and hepatitis C virus (HCV) testing among minority populations in Greater Philadelphia and New York City. J Community Health. 2024;49(4):588–97. 10.1007/s10900-023-01316-3. 38286964 10.1007/s10900-023-01316-3 PMC11974444

[30] Hu DJ, Xing J, Tohme RA, Liao Y, Pollack H, Ward JW, Holmberg SD. Hepatitis B testing and access to care among racial and ethnic minorities in selected communities across the United States, 2009–2010. Hepatology. 2013;58(3):856–62. 10.1002/hep.26286. 23359276 10.1002/hep.26286

[31] US Department of Health and Human Services. Viral Hepatitis National Strategic Plan for the United States: a roadmap to elimination for the United States, 2021–2025. Washington, DC: US Department of Health and Human Services; 2020. https://www.hhs.gov/sites/default/files/Viral-Hepatitis-National-Strategic-Plan-2021-2025.pdf.

[32] Thomas E, Cheng WH, Dylla DE, Marx SE, Carabino J, Xu Q. Awareness and epidemiology of chronic hepatitis C virus infections in Florida. Infect Dis Ther. 2022;11(1):451–62. 10.1007/s40121-021-00578-5. 34914078 10.1007/s40121-021-00578-5 PMC8847470

[33] Pinheiro PS, Jones PD, Medina H, Cranford HM, Koru-Sengul T, Bungum T, Wong R, Kobetz EN, McGlynn KA. Incidence of etiology-specific hepatocellular carcinoma: diverging trends and significant heterogeneity by race and ethnicity. Clin Gastroenterol Hepatol. 2023. 10.1016/j.cgh.2023.08.016. 37678486 10.1016/j.cgh.2023.08.016 PMC10915102

[34] US Department of Health and Human Services. (2024). Assistant Secretary for Planning and Evaluation (ASPE). Poverty Guidelines. Available at: https://aspe.hhs.gov/topics/poverty-economic-mobility/poverty-guidelines. Accessed 6 Oct 2024.

[35] R Core Team (2021). R: a language and environment for statistical computing. R Foundation for Statistical Computing, Vienna, Austria. Available at: https://www.R-project.org/. Accessed 6 Oct 2024.

[36] Dohil I, Cruz R, Tran T, Schwarz K, Gish R, Huang J. Hepatitis B screening practices at a large healthcare safety network. Hepatology. 2020;72(1 suppl):444A-A445. 10.1002/hep.31579.

[37] Sheena BS, Hiebert L, Han H, Ippolito H, Abbasi-Kangevari M, Abbasi-Kangevari Z, Abbastabar H, Abdoli A, Ali HA, Adane MM. Global, regional, and national burden of hepatitis B, 1990–2019: a systematic analysis for the Global Burden of Disease Study 2019. Lancet Gastroenterol Hepatol. 2022;7(9):796–829. 10.1016/s2468-1253(22)00124-8. 10.1016/S2468-1253(22)00124-8 PMC9349325 35738290

[38] Wong RJ, Kim D, Ahmed A, Singal AK. Patients with hepatocellular carcinoma from more rural and lower-income households have more advanced tumor stage at diagnosis and significantly higher mortality. Cancer. 2021;127(1):45–55. 10.1002/cncr.33211. 33103243 10.1002/cncr.33211

[39] Zibbell JE, Asher AK, Patel RC, Kupronis B, Iqbal K, Ward JW, Holtzman D. Increases in acute hepatitis C virus infection related to a growing opioid epidemic and associated injection drug use, United States, 2004 to 2014. Am J Public Health. 2018;108(2):175–81. 10.2105/ajph.2017.304132. 29267061 10.2105/AJPH.2017.304132 PMC5846578

[40] Pinheiro PS, Zhang J, Setiawan VW, Cranford HM, Wong RJ, Lihua L. Liver cancer etiology in Asian subgroups and American Indian, Black, Latino, and White populations. JAMA Netw Open. 2025. 10.1001/jamanetworkopen.2025.2208. 10.1001/jamanetworkopen.2025.2208 PMC11950898 40146106

[41] US Centers for Medicare and Medicaid Services. (2024). Preventive & screening services. Available at: https://www.medicare.gov/coverage/preventive-screening-services. Accessed 6 Oct 2024.

[42] Tarazi W, Welch W, Nguyen N, Bosworth A, Sheingold S, DeLew N, Sommers B: Medicare beneficiary enrollment trends and demographic characteristics. Office of the Assistant Secretary for Planning and Evaluation, US Department of Health and Human Services 2022, Issue Brief No. HP2022–08.

[43] Jaén CR, Stange KC, Nutting PA. Competing demands of primary care: a model for the delivery of clinical preventive services. J Fam Pract. 1994;38(2):166–71. 8308509

[44] Semé JL, Bivins BL, Sternberg CA, Barnett JD, Junis-Florian T, Nicolas G, Etienne M, Jean P. The Educational, health, and economic impacts of COVID-19 among Haitians in the USA: time for systemic change. J Racial Ethn Health Disparities. 2022;9(6):2171–9. 10.1007/s40615-021-01156-8. 34596889 10.1007/s40615-021-01156-8 PMC8485767

[45] Escobedo LE, Cervantes L, Havranek E. Barriers in healthcare for Latinx patients with limited English proficiency-a narrative review. J Gen Intern Med. 2023;38(5):1264–71. 10.1007/s11606-022-07995-3. 36720766 10.1007/s11606-022-07995-3 PMC9888733

[46] US Health Resources and Services Administration. Bureau of Primary Health Care. (2019). Uniform data system reporting instructions for 2019 health center data. Available at: https://bphc.hrsa.gov/sites/default/files/bphc/data-reporting/2019-uds-manual.pdf. Accessed 6 Oct 2024.

