# Supplementary material for: Chronic Viral Hepatitis Screening Inequities Across Florida Federally Qualified Health Centers
Source: J Racial Ethn Health Disparities. 2025 Mar 6;13(3):1675–84. doi: 10.1007/s40615-025-02363-3 (PMC13157420; doi:10.1007/s40615-025-02363-3)
Supplement: Supplementary file 1 — Supplementary file1 (DOCX 17 KB) [file 40615_2025_2363_MOESM1_ESM.docx]

**Title:** Chronic viral hepatitis screening inequities across Florida federally qualified health centers.

**Journal Name:** Journal of Racial and Ethnic Health Disparities

**Authors:** Hannah M. Cranford, PhD^1^; Daniel Parras, MPH^2^; Patricia D. Jones, MD, MSc^3,4^; Edelise Endemano^2^; Katherine Chung-Bridges, MD^2^; Paulo S. Pinheiro, MD, PhD^1,4^

**Affiliations:** 1: Division of Epidemiology & Population Health Sciences, Department of Public Health Sciences, University of Miami School of Medicine, Miami, FL, USA; 2: Health Choice Network, Miami, FL, USA; 3: Division of Hepatology, Department of Medicine, University of Miami School of Medicine, Miami, FL, USA; 4: Sylvester Comprehensive Cancer Center, University of Miami Health System, Miami, FL, USA.

**Corresponding Author:** Hannah Cranford; Hmc110@miami.edu.

| **Supplementary Table 1. Detailed primary language, ethnicity, and race group and by aggregate race among Health Choice Network patients. Florida, 2019–2021.** | | | | |
| --- | --- | --- | --- | --- |
| **Race, Ethnicity, and Primary Language** | **Race** | | | |
|  | **White**  **n (%)** | **Black**  **n (%)** | **Other**  **n (%)** | **Total**  **N (%)** |
| NH-White | 14,846 (27.7) | 0 (0) | 0 (0) | 14,846 (16.2) |
| Haitian Creole Speakers | X | 2,223 (8.5) | X | 2,257 (5.1) |
| English-speaking Hispanic | 11,896 (22.2) | 795 (3.1) | 2,598 (21.3) | 15,289 (16.6) |
| Spanish-speaking Hispanic | 26,754 (49.9) | 673 (2.6) | 3,472 (28.4) | 30,899 (33.6) |
| NH-Black | 0 (0) | 22,333 (85.8) | 0 (0) | 22,333 (24.3) |
| Asian | 0 (0) | 0 (0) | 1,615 (13.2) | 1,615 (18.0) |
| Other | 0 (0) | 0 (0) | 4,478 (36.6) | 4,478 (4.9) |
| **Total^a^** | 53,623 | 26,032 | 12,220 | 91,875 |
| Note: observations fewer than 10 are indicated by X. a. includes 158 Other Language Hispanic patients.  Abbreviations: N, number; NH, non-Hispanic. | | | | |
